# DNA vaccination with a gene encoding Toxoplasma gondii GRA6 induces partial protection against toxoplasmosis in BALB/c mice

**DOI:** 10.1186/1756-3305-4-213

**Published:** 2011-11-09

**Authors:** Xi-Meng Sun, Jun Zou, Elashram Saeed AA, Wen-Chao Yan, Xian-Yong Liu, Xun Suo, Heng Wang, Qi-Jun Chen

**Affiliations:** 1 National Animal Protozoa Laboratory & College of Veterinary Medicine, China Agricultural University, Beijing 100193, China; 2 College of Animal Science and Technology, Henan University of Science and Technology, Luoyang 471003, China; 3 Department of Microbiology and Parasitology, Institute of Basic Medical Sciences, Chinese Academy of Medical Sciences and School of Basic Medicine, Peking Union Medical College, Beijing 100005, China; 4 Laboratory of Parasitology, Institute of Pathogen Biology/Institute of Basic Medicine, Chinese Academy of Medical Sciences, Beijing 100730, China

## Abstract

**Background:**

Infection with the protozoan *Toxoplasma gondii *causes serious public health problems and is of great economic importance worldwide. Protection from acute toxoplasmosis is known to be mediated by CD8+ T cells, but the *T. gondii *antigens and host genes required for eliciting protective immunity have been poorly defined. The *T. gondii *dense granule protein 6 (GRA6), recently proved to be highly immunogenic and produces fully immune protection in *T. gondii *infected BALB/c mice with an H-2L^d ^gene. The CD8+ T cell response of H-2L^d ^mice infected by the *T. gondii *strain seemed to target entirely to a single GRA6 peptide HF10-H-2L^d ^complex.

**Results:**

To determine whether a GRA6-based DNA vaccine can elicit protective immune responses to *T. gondii *in BALB/c mice, we constructed a eukaryotic expression vector pcDNA3.1-HisGRA6 and tested its immunogenicity in a mouse model. BALB/c mice were vaccinated intramuscularly with three doses of GRA6 DNA and then challenged with a lethal dose of *T. gondii *RH strain tachyzoites. All immunized mice developed high levels of serum anti-GRA6 IgG antibodies, and *in vitro *splenocyte proliferation was strongly enhanced in mice adjuvanted with levamisole (LMS). Immunization with pcDNA3.1-HisGRA6 with LMS resulted in 53.3% survival of challenged BALB/c mice as compared to 40% survival of BALB/c without LMS. Additionally, immunized Kunming mice without an allele of H-2L^d ^failed to survive.

**Conclusions:**

Our result supports the concept that the acquired immune response is MHC restricted. This study has a major implication for vaccine designs using a single antigen in a population with diverse MHC class I alleles.

## Background

Infection with the intracellular parasite *Toxoplasma gondii *is responsible for toxoplasmosis in humans and other warm-blooded animals. In veterinary medicine, *T. gondii *infection has economic importance due to abortion and neonatal losses in domestic animals, or as a source of transmission to humans [1-3]. Vaccination is one of the most efficient strategies to prevent and control the spread of toxoplasmosis. A live attenuated vaccine has been developed for the prevention of chronic infection in sheep [4]. However, it cannot be used in humans because of the risk of reversion to a pathogenic form [5].

The dense granule of *T. gondii *is a secretory vesicular organelle, which produces proteins that participate in the modification of the parasitophorous vacuole (PV) and PV membrane for the maintenance of intracellular parasitism in almost all nucleated host cells [6]. There are 16 GRA proteins, GRA1-GRA10, GRA12, GRA14, 2 isoforms of nucleotide triphosphate hydrolase (NTPase I and II) [7] and 2 protease inhibitors (TgPI 1 and 2) [8,9]. All the GRA proteins are identified as excretory/secretory antigens (ESP). Several of them are suitable as DNA vaccines for immunity against toxoplasmosis. Immunization of C3H mice with a plasmid expressing granule protein 1 (GRA1) showed 75-100% protection to challenge with *T. gondii *cysts [10]. DNA vaccination with protein GRA1, GRA7, and rhoptry protein ROP2 induced protection against infection with different virulent *T. gondii *strains in C3H mice but not in BALB/c and C57BL/6 mice [11]. The GRA4 DNA vaccine containing the whole coding sequence, results in a 62% survival of susceptible C57BL/6 infected mice [12]. Intramuscular injection of sheep with a DNA liposome formulated plasmid coding for GRA1, GRA4, GRA6 and GRA7 is an effective system that induces a significant immune response against *T. gondii *[13].

Protection from acute toxoplasmosis is mediated by CD8+ T cells, but *T. gondii *antigens and host genes required for eliciting protective immunity are poorly defined [14]. The *T. gondii *dense granule protein 6 (GRA6), being highly immunogenic, is a candidate vaccine against toxoplasmosis. The HF10 peptide (HPGSVNEFDF) is located at the carboxyl terminus of GRA6 and is the immunodominant epitope to bind H-2L^d ^major histocompatibility complex class I molecule (MHC class I). It induces immune protection of BALB/c mice carrying the H-2L^d ^molecule against *T. gondii *infection. The CD8+ T cell response of BALB/c mice infected by the *T. gondii *strain seemed to be targeted entirely to the single GRA6 HF-H-2L^d ^complex [15]. Notably, similar focusing of the CD8+ T cell response to a single antigen from the circumsporozoite protein of *Plasmodium yoelii *and only a small subset of epitopes of the trans-sialidase antigens of *Trypanosoma cruzi *has been reported in mice [16,17]. DNA-based vaccination is one of the most promising strategies for the development of new generation effective vaccines against intracellular parasites.

We have constructed the eukaryotic expression vector named pcDNA3.1-HisGRA6 to determine whether DNA immunization can elicit protective immune responses to *T. gondii*. The present work shows that the partial protection against toxoplasmosis in BALB/c mice induced by DNA vaccination with *T. gondii *GRA6 gene. It has implications for vaccine designs using a single antigen in a population with diverse MHC class I alleles.

## Materials and methods

### Plasmid construction and preparation

The entire GRA6 open reading frame (ORF) was amplified by PCR from the cDNA of *T. gondii *(RH strain) tachyzoites with primer RH-1 (5'-CGCGGATCCCGCCAGCATGGCACACGGTGGCATC-3') and RH-2 (5'-GCATGCGGCCGCCTAATAATCAAACACATTCACACG-3'). The PCR product was ligated into the pGEX-4T-1 and pcDNA3.1 vectors (Invitrogen) at the BamH I and Not I sites to construct two recombinant plasmids pGEX-GRA6 and pcDNA3.1-HisGRA6. The two plasmids pGEX-GRA6 and pcDNA3.1-HisGRA6, carrying the GRA6 gene were purified from DH5α-competent cells with an endotoxin-free DNA purification kit (Vigorous Biotechnology, Beijing). The integrity of these inserts was verified by DNA sequencing and agarose gel electrophoresis after digestion with appropriate restriction enzymes. The control plasmid pcDNA3.1 was purified from the same *E. coli *strain using the same method as purifying pcDNA3.1-HisGRA6. Plasmid pGEX-GRA6 was used for expressing the GRA6 protein in bacteria and plasmid pcDNA3.1-HisGRA6 was used for immunization of mice and protein expression in mammalian cells *in vitro*. DNA for immunization was dissolved in sterile endotoxin-free PBS. The DNA concentration was determined spectrometrically at 260 nm. The OD 260/280 ratios for the purified DNA were 1.8-2.0, indicating that there was no major protein contamination.

### Mice and parasites

Six- to eight-week-old female BALB/c carrying H-2L^d ^and Kunming (without H-2L^d^) mice were purchased from the Institute of Laboratory Animal Sciences, Chinese Academy of Medical Sciences, Beijing. They were maintained in groups of six per cage with food and water provided *ad libitum *and artificial light for 12 h per day. This study was conducted in compliance with the regulations concerning the use of laboratory animals of China Agricultural University. The RH strain of *T. gondii *was maintained in our laboratory by passage of tachyzoites in BALB/c mice or Vero cells. The *T. gondii *tachyzoites used for challenge were cultured in Vero cells in DMEM medium (Sigma, USA) supplemented with 2% fetal calf serum, harvested by scrapping the cells and purified by filtration on glass fiber [18].

### Immunization

**Figure 1:**
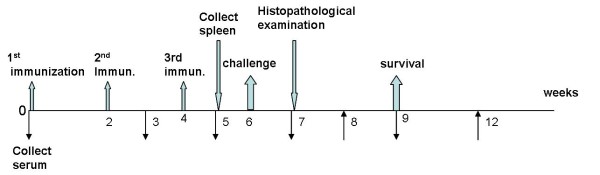
**Experimental design**.

As shown in Figure 1, BALB/c mice received three injections of 100 μg of pcDNA3.1-HisGRA6 DNA at 2-week intervals in both tibialis anterior muscles. Kunming mice were immunized with pcDNA3.1-HisGRA6 and BALB/c with empty pcDNA3.1 as controls. One group of BALB/c mice was immunized with pcDNA3.1-HisGRA6 formulated in 1% LMS as the adjuvant. The immunized mice were challenged intraperitoneally with 1, 000 tachyzoites of *T. gondii *RH strain (Type I). Sera were collected at 0, 3, 5, 7 and 9 weeks post the first immunization injection. Serum anti-GRA6 antibody levels were detected by indirect ELISA. One week post the last injection, the spleen was removed aseptically. Splenic lymphocyte proliferation was tested using the MTT assay. One week after the challenge, brain, liver and spleen were removed and fixed in 10% formalin for histological examination. Two weeks after the last immunization, the animals were observed daily for mortality.

### Expression of GRA6 in Chinese hamster ovary (CHO) cells

The plasmid pcDNA3.1-HisGRA6 or the control plasmid pcDNA3.1 were transfected into CHO cells using a commercial transfection kit (Engreen, Beijing) according to the manufacturer's instructions. CHO cells were cultured in DMEM supplemented with FBS (10% v/v), penicillin (200 U ml^-1^) and streptomycin (20 mg ml^-1^) in a humidified atmosphere of 5% CO_2 _at 37°C. The GRA6 mRNA transcription and protein expression in CHO cells were confirmed by RT-PCR and western blotting, respectively. The transfected CHO cells were processed for identification of the GRA6 mRNA transcription by RT-PCR and GRA6 protein expression by immunoblotting according to the standard technique [19]. In brief, cells were harvested 48 h after transfection, disrupted by rapid freezing and thawing 3 times using liquid nitrogen, the supernatant was obtained by centrifuging at 5, 000 g for 15 min at 4°C. Proteins in the supernatant of the samples were separated by SDS-PAGE and blotted onto a PVDF membrane, which was then blocked with 5% skimmed milk. The PVDF membrane was incubated for 1 h with murine anti-His antibodies (Tiangen, Beijing) diluted 1:4000 in 5% skimmed milk, and incubated with HRP-conjugated anti-mouse IgG (Southern Biotech, USA) diluted 1:8000 in 5% skimmed milk. The conjugated substrate was detected using a chemiluminescent dectection kit (CWBIO, China).

### Expression of GST-GRA6 protein in bacteria

The plasmid pGEX-GRA6 was transformed to *E. coli *BL21 (DE3) cells (Transgen Company, China) with kanamycin selection. The recombinants were harvested after 6 h of induction with IPTG (isopropyl β-D-1-Thiogalactopyranoside). The glutathione-S-transferase (GST)-GRA6 protein was purified using glutathione affinity chromatography.

### ELISA

Antigen-specific antibodies were quantified by enzyme-linked immuno-sorbent assay (ELISA) as previously described [20]. GST-GRA6 protein expressed in bacteria was used as the coating antigen, and antigen-specific antibodies were detected with HRP-labeled anti-mice IgG. The assay was developed using TMB. The optical density (OD) at 450 and 630 nm was measured by a plate reader (Bio-Rad, CA, USA). The serum antibody titer was defined as the highest dilution that gave a test/naive serum OD ratio of 2.1 or higher.

### Antigen-specific splenic lymphocyte proliferation

GRA6-stimulated splenic lymphocyte proliferation was used to assess the cellular immune response of the immunized mice. Three mice from each group were killed 7 days after the last immunization and the spleen was removed. Single cell suspensions were obtained by filtration through a nylon mesh. Erythrocytes in the spleen cell suspension were removed by lysis and the remaining cells were washed and suspended in RPMI 1640 medium (GIBCO) supplemented with 5% FCS, HEPES (10 mM), L-glutamine (2 mM), sodium pyruvate (1 mM), β-mercaptoethanol (50 mM), gentamycin (50 mg/ml), penicillin (100 U/ml) and streptomycin (100 mg/ml). They were seeded in triplicate in flat-bottomed 96-well microtiter plates (Costar) at 5 × 10^5 ^cells per well in 200 μl culture medium alone as the negative control or with various concentrations of GST-GRA6 fusion protein, bovine serum albumin (BSA), or 5 μg/ml concanavalin A as the positive control for 48-72 h at 37°C. Then 20 μl MTT (5 mg/ml in PBS) was added to each well. After 4 h of incubation at 41°C DMSO (100 μl/well) was added and the culture was further incubated at 37°C for 10 min to arrest cell proliferation. The optical density was measured by a plate reader at 490 nm. Data were expressed as stimulation index (SI), which is the ratio of the mean OD value of triplicate wells with antigen stimulation to the mean value of triplicate wells of the negative control [21].

### Histopathology

The formalin-fixed brain, liver and spleen tissues were dehydrated with increasing concentrations of ethanol, embedded in paraffin, sectioned and stained with hematoxylin and eosin for histological evaluation.

### Statistical analysis

Levels of significance between groups of mice were determined using the student's t-test for mortality, antibody titers and T cell proliferation stimulation index.

## Results

### Construction of DNA vaccine

**Figure 2:**
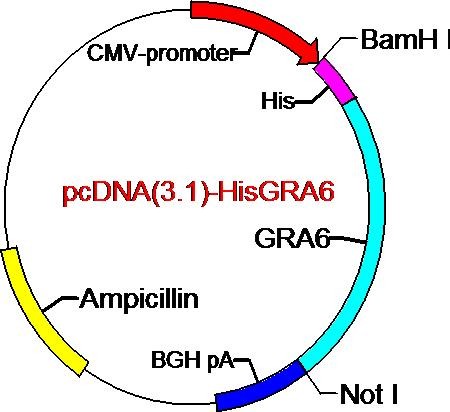
**Plasmid map of pcDNA(3.1)-GRA6**.

The GRA6 gene was cloned into the eukaryotic expression vector pcDNA3.1 using the restriction enzymes BamH I and Not I. The transcription was driven by the cytomegalovirus immediate-early promoter. This eukaryotic expression vector also contains the CpG sequence and the polyadenylation and 3' splicing signals from bovine growth hormone (Figure 2). The resulting vector consisted of 698 bases of the entire sequence of the ORF of the GRA6 gene, encoding 230 amino acids, followed by a stop codon.

### Expression of GRA6 *in vitro*

**Figure 3:**
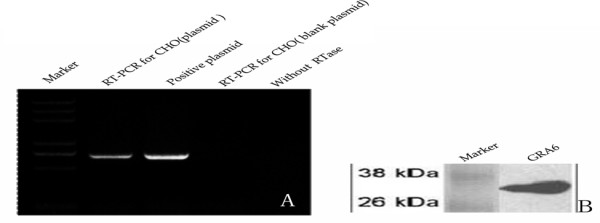
**Identification of expression of GRA6 gene in CHO cells with RT-PCR and Western blot**. A. Gel electrophoresis of RT-PCR products from pGRA6-transfected or control plasmid pcDNA3.1-transfected CHO cells; B. Western blot of GRA6 protein expressed in CHO cells.

The expression of GRA6 in a prokaryotic system and a eukaryotic system was investigated. In the prokaryotic system, plasmid pGEX-GRA6, which has high protein yield, was used for expressing GST-GRA6 fusion protein *in vitro*. In the eukaryotic system, plasmid pcDNA3.1-HisGRA6 was transfected into CHO cells. The level of mRNA and protein expression was confirmed by RT-PCR and Western blotting, respectively. Electrophoresis of the RT-PCR product from RNA of CHO transfected with pcDNA3.1-HisGRA6 showed one band of about 714 bp, corresponding to the positive plasmid control (Figure 3A). RT-PCR product from RNA of CHO transfected with the control pcDNA3.1(+) showed no similar band.

The histidine tagged GRA6 protein of 32 kDa was identified by Western blotting using anti-His monoclonal antibodies (Figure 3B).

### Humoral immune response induced by DNA vaccination

**Figure 4:**
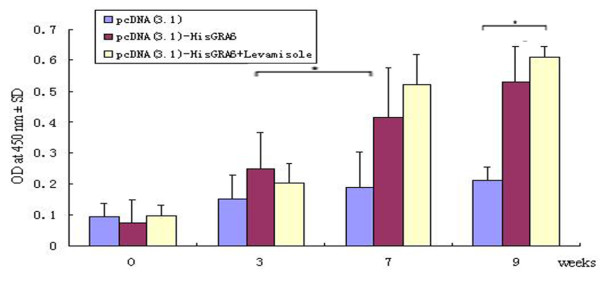
**Anti-GRA6 antibody levels in BALB/c mice (n = 15) immunized with the pcDNA3.1-HisGRA6 plasmid on days 0, 14 and 28**. One group of BALB/c mice were immunized with the empty pcDNA3.1 as the control. Sera were collected at 0, 3, 5, 7 and 9 weeks post immunization. Anti-GRA6 antibodies levels were determined by indirect ELISA. *p < 0.05

Vaccination with the plasmid DNA encoding GRA6 induced a strong antibody response. As shown in Figure 4, significant antibody responses were observed after the second immunization in BALB/c mice. While BALB/c mice were found to be good responders to the DNA vaccine after the second immunization, Kunming mice showed no immune response to the DNA vaccine (data not shown). The IgG antibody titer was greater in the sera of mice co-inoculated with LMS than in the sera of mice immunized with pcDNA3.1-HisGRA6 alone, but there was no statistically significant difference between the two groups (P > 0.05).

### Cellular immune response induced by DNA vaccination

**Figure 5:**
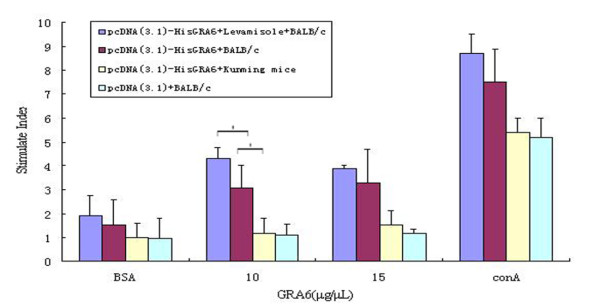
**GRA6-specific proliferation response of splenocytes from BALB/c and Kunming mice immunized with the GRA6 DNA vaccine and empty vector pcDNA3.1 vector**. The mice (n = 15/group) were immunized on days 0, 14 and 28 with 100 μg pcDNA3.1-HisGRA6. Two groups of mice, one group of Kunming mice were immunized with pcDNA3.1-HisGRA6 and one group of BALB/c mice immunized with empty pcDNA3.1 were included as controls. The level of proliferation of splenocyte T cells in spleen was tested using the MTT method assay. *p < 0.05

To evaluate anti-GRA6 immune cellular responses in the DNA-vaccinated mice, spleen cell suspensions were prepared from each mouse one week post the last immunization. Antigen specific proliferation of splenic lymphocytes was determined using the MTT assay. Significant GRA6-specific splenocyte proliferation was observed for the BALB/c mice immunized with pcDNA3.1-HisGRA6 alone or in combination with the adjuvant, LMS. The proliferation was antigen-specific since there was no expansion of splenocytes in the presence of a control protein BSA or from BALB/c mice vaccinated with the control pcDNA3.1 vector in response to GRA6 stimulation. Splenocytes from vaccinated Kunming mice did not proliferate when stimulated with GRA6 protein (Figure 5).

### Immunoprotection against lethal challenge

To determine whether the DNA vaccine could induce immunoprotection against a lethal challenge of *T. gondii*, BALB/c mice were immunized with pcDNA3.1-HisGRA6 alone, or pcDNA3.1-HisGRA6 plus LMS and challenged with *T. gondii *tachyzoites.

**Figure 6:**
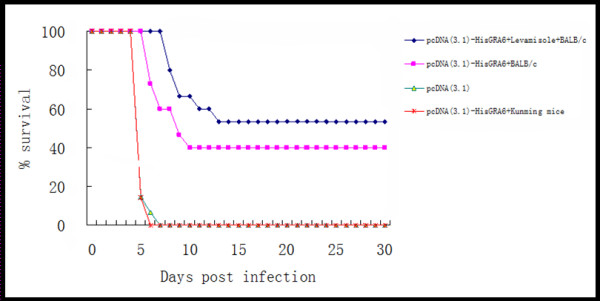
**Survival of BALB/c mice against a lethal challenge of *T. gondii***. BALB/c mice (*n *= 15/group) were immunized on days 0, 14 and 28 with 100 μg pcDNA3.1-HisGRA6 or empty pcDNA3.1 and Kunming mice with pcDNA3.1-HisGRA6. The mice were challenged with 1, 000 *T. gondii *RH strain tachyzoites 2 weeks after the last immunization, and observed daily for mortality.

As shown in Figure 6, immunization of BALB/c mice with pcDNA3.1-HisGRA6 significantly increased the survival rate of these mice (40% survival) (P < 0.05) in comparison with BALB/c immunized with the control vector or Kunming mice immunized with pcDNA3.1-HisGRA6 (0% survival) (P < 0.05). Survival was further increased in mice immunized with pcDNA3.1-HisGRA6 plus LMS (53.3% survival cf. 40% without LMS; P < 0.05). Mice alive at week 12 stayed alive for at least 2 months.

**Figure 7:**
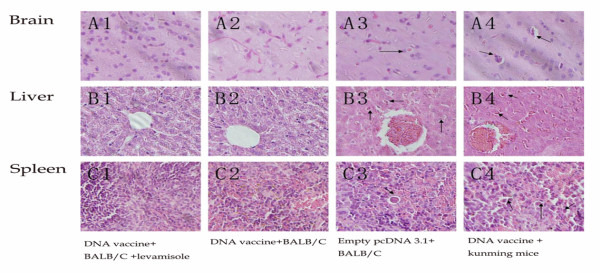
**Histopathology of brain, liver and spleen of mice (40×)**. BALB/c mice (n = 3/group) were immunized on days 0, 14 and 28 with 100 μg pcDNA3.1-HisGRA6. Two groups of mice, Kunming Mice immunized with pcDNA3.1-HisGRA6 and BALB/c immunized with empty pcDNA3.1, were included as controls. The mice were challenged intraperitoneally with 1, 000 of *T. gondii *RH strain tachyzoites. One week after the challenge, different organs (brain, liver and spleen) were collected and processed for histology. Parasites are indicated by an arrow. In this figure, A1-A4: brain; B1-B4: liver; C1-C4: spleen; A1, B1, C1: BALB/c mice immunized with pcDNA(3.1)-HisGRA6 plus LMS; A2, B2, C2: BALB/c mice immunized with pcDNA(3.1)-HisGRA6; A3, B3, C3: BALB/c mice immunized with empty pcDNA3.1; A4, B4, C4: Kunming Mice immunized with pcDNA(3.1)-HisGRA6.

Histopathological examination did not detect any parasite in the brain, liver or spleen of BALB/c mice immunized with pcDNA3.1-HisGRA6 alone or pcDNA3.1-HisGRA6 plus LMS. Parasites were clearly visible in the tissues of the control groups (Figure 7).

## Discussion

In the present study, a DNA vaccine was constructed based on the HF10 peptide sequence located at the carboxyl terminus of the GRA6 gene of *T. gondii*. The results from pcDNA3.1-HisGRA6-transfected CHO cells show that the recombinant GRA6 can be expressed in eukaryotic cells *in vitro*. Our data demonstrated that a single epitope DNA vaccine, pcDNA3.1-HisGRA6 was able to induce humoral and cellular immunity in mice and confer partial protection against acute infection of *T. gondii*. We also showed that immunogenicity of the DNA vaccine was enhanced by a non-specific chemical adjuvant, LMS. This is the first study demonstrating that a single epitope based DNA vaccine induced protective immunity against the acute *T. gondii *infection.

The immune response to *T. gondii *infection is dominated by CD8+ T cells, which is critical in the protection of hosts from intracellular parasite infection [22,23]. Resistance to toxoplasmic encephalitis has been linked to the locus encoding H-2Ld MHC class I in mice carrying the H-2L^d ^gene [24]. Blanchard *et al*. [15] found that the GRA6 protein, a polymorphic protein secreted in the parasitophorous vacuole, is an immunodominant antigen and elicited a protective CD8+ response in mice through the presentation of a single decapeptide HF10 of GRA6 by the H-2L^d ^major histocompatibility complex class I molecule. Based on the above findings, we designed a DNA vaccine expressing the HF10 fragment of GRA6, which was expected to induce strong cellular immunity and confer good protective efficacy against toxoplasmosis in BALB/c mice carrying the H-2L^d ^gene but not in Kunming mice without the H-2L^d^. Indeed, two injections of the DNA vaccine to BALB/c mice induced strong GRA6-specific cellular and humoral immunity as shown by a significant increase in serum antibodies to GRA6 and splenic lymphocyte expansion specific to GRA6 *in vitro*.

**Figure 8:**
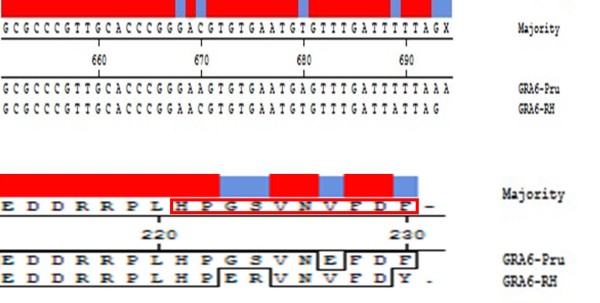
**Comparison of DNA and protein sequences of GRA6 from *T. gondii *RH (type I) and Pru (type II)**.

Immunization with the DNA vaccine conferred some protection of mice from a lethal challenge with *T. gondii *RH, but did not provide complete protection. Survival of DNA-immunized mice ranged from 40 to 53%, compared to 100% mortality of control mice immunized with the empty vector or DNA-immunized Kunming mice. The incomplete protection was possibly related to the polymorphism of the carboxy-terminal region of GRA6 in *T. gondii *strains [25]. HF10 (HPGSVNEFDF) derived from the type II strain was not only an immunodominant epitope, but was also able to elicit a protective CD8+ T cell response during the parasite infection [15]. GRA6 evolved under selective pressure, as suggested by the failure of HF10-specific T cells to recognize the corresponding GRA6 peptide from a type I strain (HPERVNVFDY). Figure 8 showed the difference of DNA and protein sequences of GRA6 from *T. gondii *RH (type I) and *T. gondii *Pru (type II).

The use of adjuvants to improve vaccine efficacy is well known, and adjuvant is used in many commercial vaccines for humans and animal species. The function of adjuvants is believed to activate innate immunity, to increase the interaction between antigen and antigen presenting cells (APC) and to improve antigen processing or presentation in APC [26]. In this study, we used a chemical adjuvant, LMS, which is quickly absorbed or metabolized by tissues at the injection site. LMS has been commonly used as an anthelmintic for domestic animals for more than 30 years and also used in several human clinical trials as an anticancer drug [27]. LMS injected with a Foot-and-Mouth Disease Virus (FMDV) DNA vaccine stimulated both humoral and cellular immune responses in conjunction with strong production of interferon (IFN)-γ [28]. We also observed some enhancement in our study, which may relate to LMS affecting several important genes within the toll-like pathway [29]. LMS might be an agonist that could activate innate response via TLR7/8 pathways. Indeed, CpG motifs [30,31], derived from the eukaryotic vector, activated innate responses via the TLR9 pathway.

## Conclusions

The DNA vaccine based on GRA6 of *T. gondii *can induce strong humoral and cellular immunity and provide partial protection against toxoplasmosis in BALB/c mice with an H-2L^d ^allele. Our result supports the notion that the immune response to *T. gondii *is MHC restricted. This study has major implications for vaccine designs using a single antigen in a population with diverse MHC class I alleles [32]. It provides a theoretical basis for the vaccine design using a single immunodominant antigen for other pathogens for which T cells are required for host protection.

## Competing interests

The authors declare that they have no competing interests.

## Authors' contributions

XMS and XS conceived and designed the study, and critically revised the manuscript. JZ, ESA, WCY and XYL participated in study design, study implementation and manuscript revision. XMS and JZ performed the experiments, analysed the data and drafted the manuscript. XYL, XS, HW and QJC helped in study implementation and data collection.

All authors read and approved the final manuscript.
